# Insufficient maternal gestational weight gain and infant neurodevelopment at 12 months of age: the Japan Environment and Children’s Study

**DOI:** 10.1007/s00431-021-04232-7

**Published:** 2021-10-12

**Authors:** Noriko Motoki, Yuji Inaba, Takumi Shibazaki, Yuka Misawa, Satoshi Ohira, Makoto Kanai, Hiroshi Kurita, Teruomi Tsukahara, Tetsuo Nomiyama, The Japan Environment and Children’s Study (JECS) Group, Michihiro Kamijima, Shin Yamazaki, Yukihiro Ohya, Reiko Kishi, Nobuo Yaegashi, Koichi Hashimoto, Chisato Mori, Shuichi Ito, Zentaro Yamagata, Hidekuni Inadera, Takeo Nakayama, Hiroyasu Iso, Masayuki Shima, Youichi Kurozawa, Narufumi Suganuma, Koichi Kusuhara, Takahiko Katoh

**Affiliations:** 1 Center for Perinatal, Environmental Epidemiology, Shinshu University School of Medicine, 3-1-1 Asahi, PediatricMatsumoto, 390-8621 Japan; 2 Division of Neuropediatrics, Nagano Children’s Hospital, Azumino, Japan; 3 Department of Pediatrics, Shinshu University School of Medicine, Matsumoto, Japan; 4 Division of Rehabilitation, Nagano Children’s Hospital, Azumino, Japan; 5 Department of Preventive Medicine and Public Health, Shinshu University School of Medicine, Matsumoto, Japan

**Keywords:** Gestational weight gain, Infant, Neurodevelopment, Developmental delay, ASQ-3

## Abstract

Abnormal maternal gestational weight gain (GWG) increases the risk of obstetric-related complications. This investigation examined the impact of GWG on infant neurodevelopmental abnormalities at 12 months of age using the data of a nationwide Japanese cohort study. Questionnaire data were obtained from the ongoing Japan Environment and Children’s Study cohort study. Maternal GWG was subdivided as below, within, or above the reference values of the Institution of Medicine pregnancy weight guidelines. The Ages and Stages Questionnaire, third edition (ASQ-3) is a parent-reported developmental screening instrument for children across five domains: communication, gross motor, fine motor, problem-solving, and personal–social. Multiple logistic regression analysis was employed to identify correlations between GWG and developmental delay defined as ASQ-3 scores of less than two standard deviations below the mean. A total of 30,694 mothers with singleton live births and partners who completed the questionnaire were analyzed. The prevalence of mothers below, within, and above the GWG guidelines was 60.4% (18,527), 32.1% (9850), and 7.5% (2317), respectively. We recorded 10,943 infants (35.7%) who were outliers in at least one ASQ-3 domain. After controlling for covariates, GWG below established guidelines was associated with a significantly higher risk of developmental delay for the communication (odds ratio [OR] 1.21, 95% confidence interval [CI] 1.09–1.34), gross motor (OR 1.14, 95% CI 1.05–1.24), fine motor (OR 1.13, 95% CI 1.04–1.24), problem-solving (OR 1.09, 95% CI 1.01–1.18), and personal–social (OR 1.15, 95% CI 1.07–1.24) domains.

*Conclusion*: This large survey revealed a possible deleterious effect of insufficient maternal GWG on infant neurodevelopment.

*Trial registration*: The Japan Environment and Children’s Study (JECS) was registered in the UMIN Clinical Trials Registry on January 15, 2018 (number UMIN000030786).

**Table:** 

**What is Known:** *• Inappropriate maternal gestational weight gain may cause obstetric complications and adverse birth outcomes.* *• Excess maternal weight gain may result in gestational diabetes, hypertension, eclampsia, caesarean delivery, and macrosomia, while insufficient maternal weight gain has been associated with pre-term birth and small for gestational age*.	
**What is New:** *• This study provides important information on a possible adverse effect of insufficient maternal gestational weight gain on offspring neurodevelopment at 12 months of age.* *• Our findings indicate a need to reconsider the optimal body mass index and gestational weight gain for women desiring pregnancy.*

**Supplementary information:**

The online version contains supplementary material available at 10.1007/s00431-021-04232-7.

## Introduction

Developmental delay is defined as delays in the areas of speech and language, motor, social, and cognitive development [1]. The incidence of developmental delay has increased dramatically in recent decades [2, 3]. Although the estimated prevalence of developmental delay is generally 5–15% in pediatric populations [2–4], reported rates vary depending on the socioeconomic characteristics of the study population, case definition, and age range [5].

Excess maternal weight gain increases the risk of obstetric complications, such as gestational diabetes, hypertensive disorder of pregnancy (HDP), eclampsia, caesarean delivery, and macrosomia [6]. On the other hand, insufficient maternal gestational weight gain (GWG) and low GWG rate have been associated with adverse birth outcomes, including pre-term birth and small for gestational age (SGA) [7, 8]. The Institute of Medicine (IOM; now known as the National Academy of Medicine) developed GWG guidelines in 1990 and later updated them in 2009 [9]. The IOM guidelines incorporate the World Health Organization (WHO) categories of maternal body mass index (BMI) and recommend lower GWG for obese women. Japan has not formally adopted the IOM guidelines, having instead developed an original set of rules for pregnancy weight management owing to limited ethnic diversity (Supplemental table S1) [10]. The Japanese guidelines are stricter for weight gain primarily to reduce obstetric complications. One large limitation of the guidelines, however, is that they lack validation from a large national study. An emerging problem in Japan is the increase in underweight pregnant women [11, 12]. Among Japanese pregnant women registered in the Japan Society of Obstetrics and Gynecology registry system, the prevalence of underweight pre-pregnancy BMI was 18.2%, versus 5.3% in the USA [12, 13]. Such a condition has been associated with an augmented risk of pre-term birth and SGA [7, 8] and possibly delayed offspring development. In Japanese women, underweight may be a larger issue than obesity.

Recent reports on the longer-term risks of maternal obesity have suggested a relationship with developmental delay in early childhood, and several epidemiologic studies have found associations between maternal obesity and various neurodevelopmental outcomes [14, 15]. In contrast, there is little evidence on the early childhood effects of maternal underweight, with none on whether excess or insufficient GWG increases the risk of offspring developmental delay. We therefore conducted a large birth cohort study with the specific objective of examining the impact of maternal GWG on early neurodevelopment.

## Materials and methods

### Study design, population, and settings

The data used in this study were obtained from the Japan Environment and Children’s Study (JECS), an ongoing cohort study that began in January 2011 to determine the effect of environmental factors on children’s health. The target number of enrolled pregnant women was 100,000. Partners were also recruited, although their participation was not mandatory. In the JECS, pregnant women were recruited between January 2011 and March 2014. The eligibility criteria for participants were as follows: (1) residing in the study area at the time of recruitment, (2) expected delivery after August 1, 2011, and (3) capable of comprehending the Japanese language and completing the self-administered structured questionnaire in Japanese. This study was registered in the UMIN Clinical Trials Registry (number UMIN000030786). Details of the JECS project have been described previously [16–18]. The JECS protocol was reviewed and approved by the Institutional Review Board on Epidemiological Studies of the Ministry of the Environment (ethical number 100910001) as well as by the ethics committees of all participating institutions. The JECS was conducted in accordance with the Helsinki declaration and other nationally valid regulations and guidelines. Written informed consent was obtained from each participant.

The present study was based on the “jecs-an-20180131” dataset released in March 2018 containing information on 98,255 mothers who had a singleton live birth, including 50,563 with the fathers’ registration. Specifically, we focused on questionnaire data regarding developmental screening as self-described by mothers when their child was 12 months old. The screening tool was the Ages and Stages Questionnaire, third edition (ASQ-3) [19]. Maternal medical information, additional pregnancy details, and medical history were collected from subject medical record transcriptions for adoption as other covariates.

### Data collection

Information on socioeconomic status, smoking habit of the mother and partner, and maternal alcohol consumption during pregnancy was collected during the second/third trimester of pregnancy (T2) by means of self-reported questionnaires. Details on a parental history of neurodevelopmental disorders, epilepsy, and mental disease were also collected from T2 questionnaires as described by the mother and partner. Maternal anthropometric data before and during pregnancy, complications and medication during pregnancy related to HDP, diabetes mellitus/gestational diabetes mellitus (DM/GDM), and neonatal information was gathered from medical records. Pre-pregnancy BMI was calculated according to WHO standards as body weight (kg)/height (m)^2^ and categorized as underweight (BMI < 18.5), normal weight (BMI 18.5–24.9), overweight (BMI 25.0–29.9), and obese (BMI ≥ 30).

#### Outcomes

The main outcomes of interest were ASQ-3 domain scores at the age of 12 months. The ASQ-3 is a parent-reported comprehensive first-level developmental screening tool for children aged 1–66 months with 30 items in five domains: communication, gross motor, fine motor, problem-solving, and personal–social skills. Each item describes a skill, ability, or behavior to which the parent responds “yes” (10 points), “sometimes” (5 points), or “not yet” (0 points). Parents sometimes omit items when they are unsure of how to respond or because they have concerns about their child’s performance of the item. ASQ-3 scores were not calculated if there were three or more omitted items in a given domain. In the case of one or two omitted items, an adjusted total domain score was calculated by adding the averaged item score either once for one omission or twice for two omissions. The score calculated for each domain was categorized as normal development (above cutoff) or referral zone (below two standard deviations). The manual for the original ASQ recommends that a child be considered as screen positive if his/her score falls below the referral cutoff in any one of the five domains [19].

**Fig. 1:**
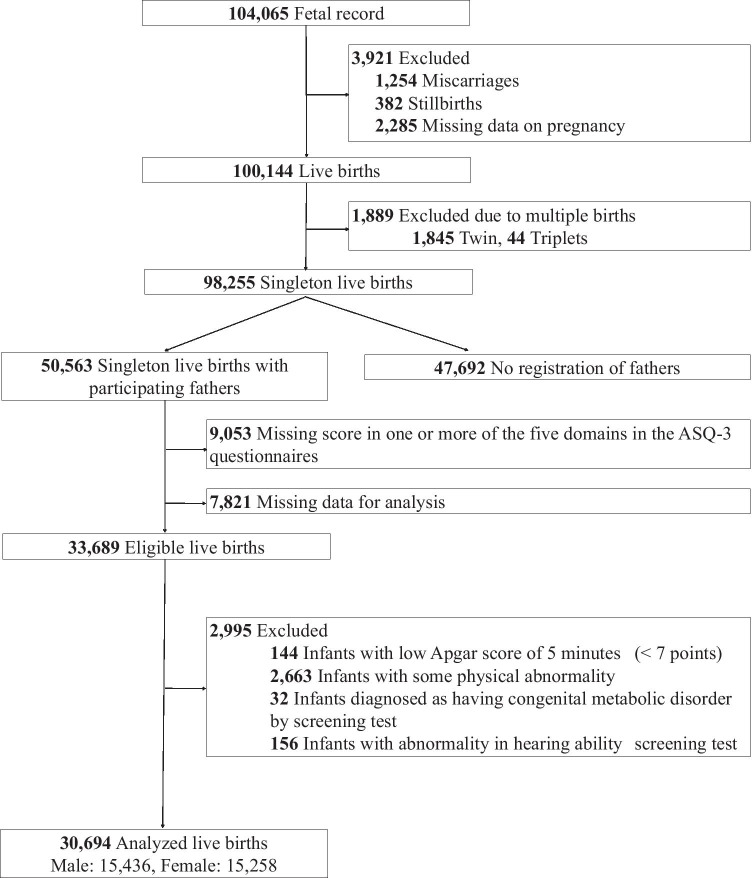
Case selection flowchart

Participants with established risk factors of developmental delay, such as neonatal asphyxia, and physical abnormality at birth, including infection, respiratory distress, congenital abnormality, hearing disability, and chromosomal abnormalities, were excluded to investigate the effects of maternal GWG on neurodevelopment in infants without obvious underlying disease during the neonatal period (Fig. 1).

#### Exposure

GWG in this study was subdivided as below, within, or above the reference values of the 2009 IOM guidelines widely used throughout the world. The IOM guideline ranges for total GWG based on pre-pregnancy BMI are as follows: 12.7–18.1 kg for underweight women, 11.3–15.9 kg for women of normal weight, 6.8–11.3 kg for overweight women, and 5.0–9.1 kg for obese women (Supplemental table S1).

#### Covariates

The covariates in our models were selected a priori based on previous literature and biologic plausibility [20–24]. We estimated the effects of GWG after adjusting for demographic data including maternal age, pre-pregnancy BMI, parental smoking habit, maternal drinking habit, maternal highest level of education, annual household income, parental history of neurodevelopmental disorders, epilepsy, and mental disease, as well as obstetric and medical variables such as parity, means of pregnancy (including spontaneous pregnancy and assisted reproductive techniques, such as ovulation induction and artificial insemination or in vitro fertilization), use of folic acid supplements, complications during pregnancy (including DM/GDM, HDP, and intrauterine growth restriction), means of delivery, birth weight, gender, method of feeding, and neonatal jaundice in the newborn period requiring treatment such as phototherapy and exchange transfusion. Parental medical history of neurodevelopmental disorders included attention deficit hyperactivity disorder, learning disability, autism, Asperger’s syndrome, pervasive developmental disorder, and others. Parental history of mental disease included depression, schizophrenia, and anxiety disorder. Intrauterine growth restriction was defined as estimated fetal weight less than −1.5 standard deviations of standard weight based on gestational age in Japan.

### Statistical analysis

Distribution normality was confirmed by the Kolmogorov–Smirnov test. Data are expressed as the mean ± standard deviation or the median (interquartile range) depending on whether they are normally distributed or not. Possible differences in maternal age, pre-pregnancy BMI, GWG, gestational age, and birth weight between subjects with normal development and developmental delay were assessed by the unpaired *t*-test or the Mann–Whitney *U* test based on the presence or absence of normal distribution, respectively. We also categorized continuous and ordinal variables, such as maternal age (< 35 or ≥ 35 years), pre-pregnancy BMI, GWG (below, within, or above), annual household income (< 4,000,000, 4,000,000–7,999,999, or ≥ 8,000,000 JPY), gestational age (< 37 or ≥ 37 weeks), and birth weight (< 1500, 1500–2499, or ≥ 2500 g). Fisher’s exact tests or chi-square tests were performed to compare covariates between groups stratified by category as well as by the presence of developmental delay. Additionally, differences in the scores of each domain among the three GWG groups were assessed by one-way repeated measures of analysis of variance (ANOVA) followed by post hoc (Bonferroni) testing. We employed multiple logistic regression models to investigate developmental delay at 1 year as the dependent variable in association with maternal GWG. Infants below and above the cutoff for each domain were categorized as “delayed” and “normal,” respectively. GWG was subdivided as below, within (reference), or above IOM guidelines. The models were adopted to calculate adjusted odds ratios (ORs) and their 95% confidence intervals (CIs) controlling covariates, as described above. Spearman’s rank correlation coefficient was used to check for multicollinearity of covariates. The variable of gestational age was excluded from the covariates because it was multicollinear with birth weight. Hosmer–Lemeshow testing was used to assess the goodness of fit of the models. We also analyzed the subjects without registered fathers to evaluate for possible selection bias.

All statistical analyses were performed using SPSS statistical software version 27 (SPSS Inc., Chicago, IL). All tests were two-tailed, and *P*-values of less than 0.05 were considered to indicate statistical significance.

## Results

A total of 30,694 mothers with singleton live births and partners who completed the JECS questionnaire were available for analysis (Fig. 1). According to the pre-pregnancy BMI categories, the prevalence of underweight, normal weight, overweight, and obese mothers was 15.4% (4730), 74.2% (22,761), 8.1% (2485), and 2.3% (718), respectively. The prevalence of mothers below, within, and above the IOM-based GWG guidelines was 60.4% (18,527), 32.1% (9850), and 7.5% (2317), respectively. There were 10,943 participants (35.7%) who were outliers in at least one ASQ-3 domain (Table 1 and Supplemental table S2).

**Table 1:** Demographic characteristics of participants with or without developmental abnormality

Variable	Total participants	Normal development	Positive ASQ-3 screen ≥ 1 domain	*P* value*
Participants, *n*	30,694	19,751	10,943	
Pre-pregnancy BMI, kg/m^2^	20.6 (19.1, 22.5)	20.6 (19.1, 22.5)	20.5 (19.1, 22.6)	0.61^†^
Pre-pregnancy BMI group, *n* (%)				0.160
Underweight (BMI < 18.5)	4730 (15.4)	2995 (15.2)	1735 (15.9)	
Normal weight (BMI 18.5–24.9)	22,761 (74.2)	14,721 (74.5)	8040 (73.5)	
Overweight (BMI 25.0–29.9)	2485 (8.1)	1590 (8.1)	895 (8.2)	
Obese (BMI ≥ 30.0)	718 (2.3)	445 (2.3)	273 (2.5)	
Maternal GWG, kg	10.2 (8.0, 12.5)	10.4 (8.1, 12.8)	9.9 (7.7, 12.2)	< 0.001^†^
Maternal GWG group, *n* (%)				< 0.001
Below	18,527 (60.4)	11,567 (58.6)	6960 (63.6)	
Within	9850 (32.1)	6575 (33.3)	3275 (29.9)	
Above	2317 (7.5)	1609 (8.1)	708 (6.5)	
Maternal age at delivery, years	31 (28, 35)	31 (28, 34)	32 (29, 35)	< 0.001^†^
Maternal age group, *n* (%)				< 0.001
< 35 years	20,463 (66.7)	13,647 (69.1)	6816 (62.3)	
≥ 35 years	10,231 (33.3)	6104 (30.9)	4127 (37.7)	
Highest level of maternal education, *n* (%)				< 0.001
Junior high school	1020 (3.3)	735 (3.7)	285 (2.6)	
High school	9094 (29.6)	5932 (30.0)	3162 (28.9)	
Vocational school/junior college	13,366 (43.5)	8670 (43.9)	4696 (42.9)	
University/graduate school	7214 (23.5)	4414 (22.3)	2800 (25.6)	
Annual household income,^‡^ *n* (%)				0.001
< 4,000,000 JPY	11,894 (38.8)	7796 (39.5)	4098 (37.4)	
4,000,000–7,999,999 JPY	15.503 (50.5)	9893 (50.1)	5610 (51.3)	
≥ 8,000,000 JPY	3297 (10.7)	2062 (10.4)	1235 (11.3)	
Maternal smoking during pregnancy, *n* (%)	1037 (3.4)	741 (3.8)	296 (2.7)	< 0.001
Partner’s smoking during pregnancy, *n* (%)	12,812 (41.7)	8657 (43.8)	4155 (38.0)	< 0.001
Maternal drinking during pregnancy, *n* (%)	568 (1.9)	366 (1.9)	202 (1.8)	0.97
Maternal history of mental disease, *n* (%)	1567 (5.1)	996 (5.0)	571 (5.2)	0.50
Maternal history of developmental disorder, *n* (%)	14 (0.05)	5 (0.03)	9 (0.08)	0.046
Maternal history of epilepsy, *n* (%)	158 (0.5)	87 (0.4)	71 (0.6)	0.015
Partner’s history of mental disease, *n* (%)	753 (2.5)	462 (2.3)	291 (2.7)	0.083
Partner’s history of developmental disorder, *n* (%)	21 (0.07)	13 (0.07)	8 (0.07)	0.82
Partner’s history of epilepsy, *n* (%)	123 (0.4)	72 (0.4)	51 (0.5)	0.19

*ASQ-3* Ages and Stages Questionnaire, third edition, *BMI* body mass index, *GWG* gestational weight gain, *JPY* Japanese yen

^*^P value for normal development versus positive screen

^†^Mann–Whitney *U* test of normal development versus positive screen. Continuous variables are expressed as the median (interquartile range)

^‡^The average (median) annual Japanese household income in 2018 was 5,523,000 JPY (4,370,000 JPY). The currency exchange rates on July 12, 2021, were 1 USD = 110 JPY and 1 EUR = 130 JPY

Table 1 and Supplemental table S2 summarize the participants’ characteristics and offspring outcomes for developmental delay. There were significant differences in the rates of the GWG groups. We observed significant differences between the normal development and developmental delay groups for demographic categories including maternal age, maternal educational level, annual household income, parental smoking status, and maternal history of epilepsy (Table 1). Significant differences were also seen in such perinatal categories as parity, means of pregnancy of current birth, maternal use of folic acid supplements, HDP, mode of delivery, gestational age, birth weight, gender, method of feeding, and neonatal jaundice (Supplemental table S2).

**Fig. 2:**
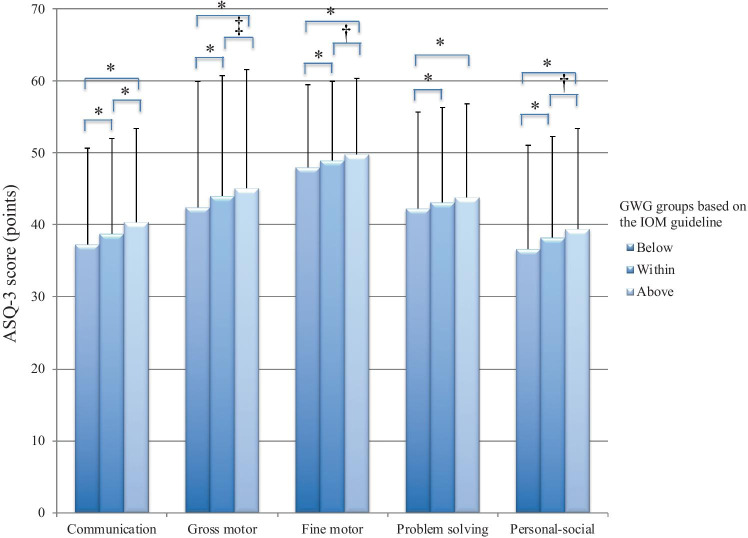
Comparison of ASQ-3 scale scores according to maternal gestational weight gain. *P < 0.001, ^†^*P* < 0.01, ^‡^*P* < 0.05

ASQ-3 domain classifications and proportions of a risk of developmental delay at 12 months according to maternal GWG are shown in Table 2 and Fig. 2. Chi-square analysis revealed significant differences in the prevalence of developmental delay in the communication, gross motor, fine motor, problem-solving, and personal–social domains among maternal GWG groups. ANOVA showed that the scores for every ASQ-3 domain were significantly lower in the GWG below guidelines group than in the GWG within and above guidelines groups.

**Table 2:** ASQ-3 domain scores and proportions at risk of delay according to maternal gestational weight gain

ASQ-3 domain (cutoff score)	Below	Within	Above	*P* value
	*n* = 18,527	*n* = 9850	*n* = 2317	
Communication (15.64 points)				
Score (points)	37.3 ± 13.4	38.7 ± 13.2*	40.3 ± 13.0*^,†^	< 0.001
On track, *n* (%)	17,141 (92.5)	9266 (94.1)	2211 (95.4)	
Referral, *n* (%)	1386 (7.5)	584 (5.9)	106 (4.6)	< 0.001
Gross motor (21.49 points)				
Score (points)	42.4 ± 17.5	44.0 ± 16.7*	45.1 ± 16.5*^,§^	< 0.001
On track, *n* (%)	15,833 (85.5)	8652 (87.8)	2056 (88.7)	
Referral, *n* (%)	2694 (14.5)	1198 (12.2)	261 (11.3)	< 0.001
Fine motor (34.50 points)				
Score (points)	48.0 ± 11.5	48.9 ± 11.0*	49.8 ± 10.6*^,‡^	< 0.001
On track, *n* (%)	16,600 (89.6)	8977 (91.1)	2147 (92.7)	
Referral, *n* (%)	1927 (10.4)	873 (8.9)	170 (7.3)	< 0.001
Problem-solving (27.32 points)				
Scores (points)	42.2 ± 13.4	43.1 ± 13.2*	43.8 ± 13.0*	< 0.001
On track, *n* (%)	15,633 (84.4)	8437 (85.7)	2023 (87.3)	
Referral, *n* (%)	2894 (15.6)	1413 (14.3)	294 (12.7)	< 0.001
Personal–social (21.73 points)				
Scores (points)	36.6 ± 14.4	38.2 ± 14.1*	39.4 ± 14.0*^,‡^	< 0.001
On track, *n* (%)	15,225 (82.2)	8399 (85.3)	2006 (86.6)	
Referral, *n* (%)	3302 (17.8)	1451 (14.7)	311 (13.4)	< 0.001

Plus-minus variables are the mean ± standard deviation. Differences in scores of ASQ-3 domains were assessed with one-way repeated measures of variance (ANOVA) followed by post hoc (Bonferroni) testing

*ASQ-3* Ages and Stages Questionnaire, third edition

^*^P < 0.001 versus the GWG below guidelines group; ^†^*P* < 0.001, ^‡^*P* < 0.01, and ^§^*P* < 0.05 versus the GWG within guidelines group

The regression models for all domains demonstrated good fit in Hosmer–Lemeshow testing. In multivariate logistic regression analysis after adjustment for covariates, compared with ideal GWG, GWG below guidelines was significantly associated with a higher incidence of developmental delay in the communication (OR 1.21, 95% CI 1.09–1.34), gross motor (OR 1.14, 95% CI 1.05–1.24), fine motor (OR 1.13, 95% CI 1.04–1.24), problem-solving (OR 1.09, 95% CI 1.01–1.18), and personal–social (OR 1.15, 95% CI 1.07–1.24) domains (Table 3). For every 2.3 kg (5 lb) of GWG, the risk of abnormalities was reduced by 4–9% in each domain of ASQ-3 (communication, OR 0.91 [95% CI 0.88–0.94]; gross motor, OR 0.96 [95% CI 0.94–0.98]; fine motor, OR 0.94 [95% CI 0.91–0.96]; problem-solving, OR 0.95 [0.93–0.97]; personal–social, OR 0.94 [0.92–0.96]) (Table 3).

**Table 3:** Odds ratios and 95% confidence intervals for the association between gestational weight gain (GWG) categories and developmental delay in ASQ-3 domains

ASQ-3 domain	Within GWG (reference)	Below GWG		Above GWG		Every 2.3-kg (5-lb) increase
	No. cases/normal development	No. cases/normal development	OR (95% CI)	No. cases/normal development	OR (95% CI)	OR (95% CI)
Communication	584/6575	1386/11,567	1.21 (1.09–1.34)	106/1609	0.82 (0.66–1.03)	0.91 (0.88–0.94)
Gross motor	1198/6755	2694/11,567	1.14 (1.05–1.24)	261/1609	0.98 (0.84–1.13)	0.96 (0.94–0.98)
Fine motor	873/6575	1927/11,567	1.13 (1.04–1.24)	170/1609	0.84 (0.70–1.00)	0.94 (0.91–0.96)
Problem-solving	1413/6575	2894/11,567	1.09 (1.01–1.18)	294/1609	0.85 (0.74–0.98)	0.95 (0.93–0.97)
Personal–social	1451/6575	3302/11,567	1.15 (1.07–1.24)	311/1609	0.94 (0.82–1.08)	0.94 (0.92–0.96)

ORs were adjusted for maternal age, pre-pregnancy BMI, parental smoking habit, maternal drinking habit, maternal highest level of education, annual household income, parental history of developmental disorders, epilepsy, and mental disease, means of pregnancy, use of folic acid supplements, complications during pregnancy (including DM/GDM and HDP), intrauterine growth restriction, gender, birth weight, method of feeding, and neonatal jaundice

*ASQ-3* Ages and Stages Questionnaire, third edition, *OR* odds ratio, *CI* confidence interval, *GWG* gestational weight gain, *BMI* body mass index, *DM/GDM* diabetes mellitus/gestational diabetes mellitus, *HDP* hypertensive disorder of pregnancy

**Fig. 3:**
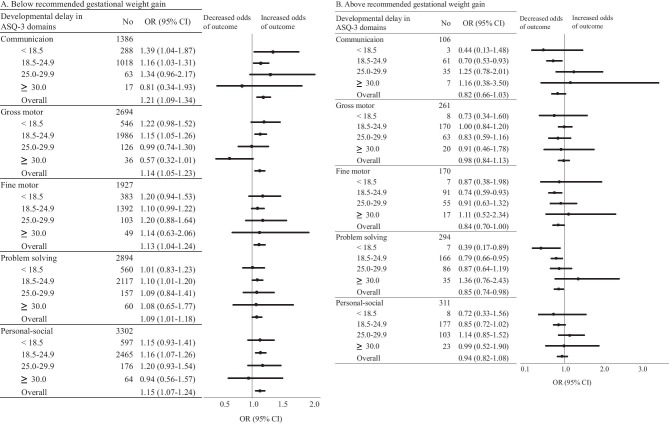
Odds ratios (ORs) for the association between maternal gestational weight gain (GWG) below and above guidelines with developmental delay in ASQ-3 domains according to the pre-pregnancy body mass index (BMI) categories. ORs are shown for the association between GWG below (**A**) and above (**B**) guidelines with developmental delay in ASQ-3 domains. The reference group is mothers with the recommended weight gain in each category of pre-pregnancy BMI. These ORs were adjusted for maternal age, pre-pregnancy BMI, parental smoking habit and maternal drinking habit during pregnancy, maternal highest level of education, annual household income, parental history of developmental disorders, epilepsy, mental disease, means of pregnancy, use of folic acid supplements, complications during pregnancy (including DM/GDM and HDP), intrauterine growth restriction, gender, birth weight, method of feeding, and neonatal jaundice

Across BMI categories, GWG below guidelines tended to associate with a higher risk of developmental delay (i.e., OR > 1.0) in ASQ-3 screening than did GWG within guidelines (Fig. 3A). In contrast, GWG above guidelines often tended to associate with a lower risk of developmental delay across domains as compared with GWG within guidelines (Fig. 3B).

Lastly, we analyzed the 24,823 subjects without registered fathers. Supplemental table S2 shows the characteristics of the normal development and developmental delay groups. We observed a significant difference in the proportion of GWG categories between the groups similar to that in the main analysis (Supplemental table S3). Multivariate regression analysis also revealed significant associations between GWG below guidelines and the incidence of developmental delay in all five domains. For every 2.3 kg (5 lb) of GWG, the risk of abnormalities was reduced by 5–11% in each domain of ASQ-3 (Supplemental table S4).

## Discussion

We herein describe the first large-scale nationwide birth cohort study in Japan to clarify the impact of insufficient maternal weight gain during pregnancy on offspring neurodevelopment at 12 months. Across pre-pregnancy BMI categories, the association was particularly significant in mothers with a lower pre-pregnancy BMI.

In this Japanese nationwide birth cohort study, the prevalence rate of screen positive measured by ASQ-3 at 12 months of age for communication, gross motor, fine motor, problem-solving, and personal–social domains was 6.8%, 13.5%, 9.7%, 15.0%, and 16.5%, respectively. However, the prevalence of developmental delay can differ according to demographic status and underlying disease [4, 5, 20–22]. Several perinatal risk factors of developmental delay have been reported, including pre-term birth, perinatal maternal mental health, and maternal educational level [20–22]. Relationships between maternal obesity during pregnancy and poor pregnancy results have also been described [6, 7, 14, 15]. The number of underweight pregnant women in Japan is on the rise [11, 12, 25, 26], possibly since the ideal body shape of young women is becoming thinner [27] in addition to strict GWG management to facilitate delivery without obstetric complications [28]. The obesity classification and GWG recommendations used in Japan differ considerably from those prescribed by the IOM (Supplemental table S1) [10]. Several recent Japanese studies showed that underweight women carried a higher risk of adverse birth outcomes, such as pre-term birth and SGA [25, 26, 29]. However, they did not assess subsequent neurodevelopment in infants of underweight women. A Swedish cohort study investigating the association between maternal GWG and risk of offspring autism spectrum disorder (ASD) supported our findings, whereby an elevated risk of ASD was observed for both insufficient and excess GWG [30]. They also suggested that maternal undernutrition during pregnancy contributed to the risk developmental abnormality.

It is uncertain why insufficient GWG may cause neurodevelopmental disorders. One reason is that malnutrition may restrict fetal brain growth. In Japan, total calorie intake among pregnant women was far below nationally recommended levels [31, 32]. Maternal dietary quality is of critical importance since specific nutrients are required during sensitive or critical periods of fetal development [33]. Folic acid has been recognized as necessary for neural tube development [34]. Iron is the most common nutrient deficiency during pregnancy and is necessary for myelination and the development of the frontal cortex and basal ganglia [35]. The studies on Japanese pregnant women mentioned above reported that the proportions of carbohydrates and lipids in total calories were respectively lower and higher than those required by pregnant women [31, 32]. Sussman et al. suggested that prenatal exposure to a carbohydrate-restricted diet, such as recently popular ketogenic diet programs, influenced not only offspring neuroanatomy such as brain structure and volumetric change [36], but also behavioral alterations that included reduced susceptibility to anxiety and depression and elevated hyperactivity in adult mouse offspring [36, 37]. Indeed, optimal diet and weight gain guidance for underweight women of child-bearing age appear critical.

It is important to determine whether neurodevelopmental evaluations at 12 months are clinically valid for subsequent diagnosis. In one study longitudinally comparing child ASQ-3 domain screening results based on cutoff scores, the vast majority (88.9–96.7%) received the same categorization results at 9, 18, and 24 months of age [38]. Other studies have provided evidence on the concurrent validity of the ASQ-3 and the clinical diagnosis of developmental delay, as well as on the reliability of the ASQ-3 in a multiethnic population [39–41]. However, the number of children who were screen positive (i.e., failed at least one of the five domains) in this study was high at 35.7%. This rate varies among age, developmental area, and country at 13–48% [38–42]. One report that evaluated the validity of the Japanese translation of ASQ-3 suggested an alternative deficit criterion of failure in at least two domains [43]. In the present investigation, the majority of screen-positive children had a failure in one domain, which could have been an overestimate; to verify this, the study cohort will be followed until the age of 13 years.

A strength of this investigation was that not only maternal, but also paternal history of neurodevelopmental problems was adjusted for as covariates. Genetic influences could be larger than those of a shared environment on the incidence of neurodevelopmental disorders [23, 24]. Since selection bias might have been produced by excluding the subjects without father registration, we also analyzed the group without father registration to assess this possibility. GWG below guidelines was significantly associated with a higher incidence of developmental delay than in the main analysis, although paternal medical history was not adjusted as a covariate in this subpopulation (Supplemental table S3).

This study has several limitations. First, the data regarding developmental scores as measured by ASQ-3 were collected from parental self-reported questionnaires and therefore subjective. Second, as data on abnormalities were evaluated at 12 months of age, no neurodevelopmental disorders diagnosed afterwards were included. Third, the large attrition rate of either unpaired participants or those not completing the ASQ-3 questionnaire may have constituted selection bias; we cannot conclusively rule out the possibility of underreporting the incidence of developmental disorders. Fourth, the parental histories of neurodevelopmental disorders, epilepsy, and mental disease were also collected from self-reported questionnaires. Therefore, these answers might not have conformed to diagnostic criteria or ICD coding. Finally, the participants of this study contained a large group of underweight mothers, which was representative of the Japanese population [12]. Therefore, although the analysis of obesity and/or excessive GWG may have been inadequate, this study provides valuable and unique research that is impossible in other countries.

Despite the above limitations, this is the first investigation using a large dataset from a Japanese nationwide birth cohort study to examine the independent influence of insufficient maternal GWG on offspring’s neurodevelopment that controlled for confounders identified by previous reports including birth weight. This study indicates a need to reconsider the optimal BMI and GWG for women desiring pregnancy not only in Japan, but also in other developed countries.

## Supplementary Information

Below is the link to the electronic supplementary material.

Supplementary file1 (DOCX 37 KB)

Supplementary file2 (XLSX 22 KB)

## Data Availability

Data are unsuitable for public deposition due to ethical restrictions and legal framework of Japan. It is prohibited by the Act on the Protection of Personal Information (Act No. 57 of 30 May 2003, amendment on 9 September 2015) to publicly deposit the data containing personal information. Ethical Guidelines for Medical and Health Research Involving Human Subjects enforced by the Japan Ministry of Education, Culture, Sports, Science and Technology and the Ministry of Health, Labour and Welfare also restricts the open sharing of the epidemiologic data. All inquiries about access to data should be sent to jecs-en@nies.go.jp. The person responsible for handling enquiries sent to this e-mail address is Dr. Shoji F. Nakayama, JECS Programme Office, National Institute for Environmental Studies.

## Abbreviations

ANOVA: Analysis of variance
ASD: Autism spectrum disorder
ASQ-3: Ages and Stages Questionnaire, third edition
BMI: Body mass index
CI: Confidence interval
DD: Developmental delay
DM/GDM: Diabetes mellitus/gestational diabetes mellitus
GWG: Gestational weight gain
HDP: Hypertensive disorders of pregnancy
IOM: Institute of Medicine
JECS: Japan Environment and Children’s Study
OR: Odds ratio
SGA: Small for gestational age
WHO: World Health Organization
