# Supplementary material for: Insufficient maternal gestational weight gain and infant neurodevelopment at 12 months of age: the Japan Environment and Children’s Study
Source: Eur J Pediatr. 2021 Oct 12;181(3):921–31. doi: 10.1007/s00431-021-04232-7 (PMC8897327; doi:10.1007/s00431-021-04232-7)
Supplement: Supplementary file 1 — Supplementary file1 (DOCX 37 KB) [file 431_2021_4232_MOESM1_ESM.docx]

| **Supplemental table S1. GWG recommendations based on 2009 IOM [9] and Japanese recommendations [10]** | | | |  |
| --- | --- | --- | --- | --- |
|  | Underweight | Normal weight | Overweight | Obese |
| Pre-pregnancy BMI (kg/m^2^) | < 18.5 | 18.5-24.9 | 25.0-29.9 | ≥ 30 |
| Total weight gain range by IOM (kg) | 12.7-18.1 | 11.3-15.9 | 6.8-11.3 | 5.0-9.1 |
| Total weight gain range by Japanese recommendations (kg) | 9.0-12.0 | 7.0-12.0 | Approximately 5.0 (tailored to the woman) | |

GWG, gestational weight gain; IOM, Institute of Medicine; BMI, body mass index.

| **Supplemental table S2. Perinatal characteristics of participants with or without developmental abnormality** | | | | | |
| --- | --- | --- | --- | --- | --- |
| Variable | | Total participants | Normal development | Positive ASQ-3 screen ≥ 1 domain | P value ^*^ |
| Participants, n | | 30,694 | 19,751 | 10,943 |  |
| Parity, n (%) | |  |  |  |  |
|  | Multiparous | 16,969 (55.3) | 11,132 (56.4) | 5,837 (53.3) | < 0.001 |
| Means of pregnancy for current birth, n (%) | |  |  |  | < 0.001 |
|  | Spontaneous | 28,591 (93.1) | 18,548 (93.9) | 10,043 (91.8) |  |
|  | Ovulation induction through medication | 814 (2.7) | 493 (2.5) | 321 (2.9) |  |
|  | Artificial insemination or *in vitro* fertilization | 1,289 (4.2) | 710 (3.6) | 579 (5.3) |  |
| Maternal use of folic acid supplements, n (%) | | 698 (2.3) | 422 (2.1) | 276 (2.5) | 0.030 |
| Diabetes mellitus/gestational diabetes mellitus, n (%) | | 879 (2.9) | 542 (2.7) | 337 (3.1) | 0.091 |
| Hypertensive disorder of pregnancy, n (%) | | 858 (2.8) | 503 (2.5) | 355 (3.2) | < 0.001 |
| Intrauterine growth restriction, n (%) | | 516 (1.7) | 321 (1.6) | 195 (1.8) | 0.31 |
| Mode of delivery for current birth, n (%) | |  |  |  | < 0.001 |
|  | Spontaneous delivery | 17,823 (58.1) | 11,731 (59.4) | 6,092 (55.7) |  |
|  | Induced delivery | 5,575 (18.2) | 3,649 (18.5) | 1,926 (17.6) |  |
|  | Vacuum extraction/Forceps delivery | 1,999 (6.5) | 1,271 (6.4) | 728 (6.7) |  |
|  | Cesarean section | 5,297 (17.3) | 3,100 (15.7) | 2,197 (20.1) |  |
| Gestational age (weeks) | | 39 (38, 40) | 39 (38, 40) | 39 (38, 40) | < 0.001 ^†^ |
| Gestational age, n (%) | |  |  |  | < 0.001 |
|  | < 37 weeks | 554 (1.8) | 296 (1.5) | 258 (2.4) |  |
|  | ≥ 37 weeks | 30,140 (98.2) | 19,455 (98.5) | 10,685 (97.6) |  |
| Birth weight, g | | 3044 (2810, 3290) | 3060 (2832, 3308) | 3010 (2776, 3256) | < 0.001 ^†^ |
| Birth weight, n (%) | |  |  |  | < 0.001 |
|  | < 1500 g | 0 | 0 | 0 |  |
|  | 1500-2499 g | 1,720 (5.6) | 949 (4.8) | 771 (7.0) |  |
|  | ≥ 2500 g | 28,974 (94.4) | 18,802 (95.2) | 10,172 (93.0) |  |
| Gender (male), n (%) | | 15,436 (50.3) | 9,506 (48.1) | 5,930 (54.2) | < 0.001 |
| Method of feeding, n (%) | |  |  |  | < 0.001 |
|  | Breast feeding | 16,809 (54.8) | 11,147 (56.4) | 5,662 (51.7) |  |
|  | Mixed feeding | 12,652 (41.2) | 7,889 (39.9) | 4,763 (43.5) |  |
|  | Infant formula | 1,001 (3.2) | 583 (3.0) | 418 (3.8) |  |
|  | Other | 232 (0.8) | 132 (0.7) | 100 (0.9) |  |
| Neonatal jaundice, n (%) | | 4,232 (13.8) | 2,639 (13.4) | 1,593 (14.6) | 0.004 |
| Total number of positive ASQ-3 domains, n (%) | |  |  |  | N.A. |
|  | 0 domains | 19,751 (64.3) | 19,751 (100) | - |  |
|  | 1 domain | 6,133 (20.0) | - | 6,133 (56.0) |  |
|  | 2 domains | 2,727 (8.9) | - | 2,727 (24.9) |  |
|  | 3 domains | 1,280 (4.2) | - | 1,280 (11.7) |  |
|  | 4 domains | 578 (1.9) | - | 578 (5.3) |  |
|  | 5 domains | 225 (0.7) | - | 225 (2.1) |  |

ASQ-3, Ages and Stages Questionnaire, third edition; N.A., not available.

^*^ P value for Normal development vs. Positive screen.

^†^ Mann–Whitney U test of Normal development vs. Positive screen. Continuous variables are expressed as the median (interquartile range).

| **Supplemental table S3. Characteristics of participants without registered father based on the presence of a developmental abnormality** | | | | |
| --- | --- | --- | --- | --- |
| Variable | | Normal development | Positive ASQ-3 screen ≥ 1 domain | P value |
| Participants, n | | 15,964 | 8,859 |  |
| Pre-pregnancy BMI, kg/m^2^ | | 20.5 (19.1, 22.4) | 20.4 (19.0, 22.3) | 0.16 ^*^ |
| Pre-pregnancy BMI group, n (%) | |  |  | 0.70 |
|  | Underweight (BMI < 18.5) | 2,542 (15.9) | 1,449 (16.4) |  |
|  | Normal weight (BMI 18.5-24.9) | 11,884 (74.4) | 6,588 (74.4) |  |
|  | Overweight (BMI 25.0-29.9) | 1,198 (7.5) | 640 (7.2) |  |
|  | Obese (BMI ≥ 30.0) | 340 (2.1) | 182 (2.1) |  |
| Maternal GWG, kg | | 10.5 (8.2, 12.9) | 10.0 (7.8, 12.3) | < 0.001 ^*^ |
| Maternal GWG group, n (%) | |  |  | < 0.001 |
|  | Below | 9,159 (57.4) | 5,653 (63.8) |  |
|  | Within | 5,426 (34.0) | 2,628 (32.6) |  |
|  | Above | 1,379 (8.6) | 578 (6.5) |  |
| Maternal age at delivery, years | | 31.2 (28.0, 35.0) | 32.0 (29.0, 36.0) | < 0.001 ^*^ |
| Maternal age group, n (%) | |  |  | < 0.001 |
|  | < 35 years | 11,655 (73.0) | 5,961 (67.3) |  |
|  | ≥ 35 years | 4,309 (27.0) | 2,898 (32.7) |  |
| Highest level of maternal education, n (%) | |  |  | < 0.001 |
|  | Junior high school | 774 (4.8) | 287 (3.2) |  |
|  | High school | 5,080 (31.8) | 2,603 (29.4) |  |
|  | Vocational school/Junior college | 6,769 (42.4) | 4,766 (42.5) |  |
|  | University/Graduate school | 3,341 (20.9) | 2,203 (24.9) |  |
| Annual household income ^†^, n (%) | |  |  | 0.001 |
|  | < 4,000,000 JPY | 6,587 (41.3) | 3,464 (39.1) |  |
|  | 4,000,000-7,999,999 JPY | 7,684 (48.1) | 4,374 (49.4) |  |
|  | ≥ 8,000,000 JPY | 1,693 (10.6) | 1,021 (11.5) |  |
| Maternal smoking during pregnancy, n (%) | | 749 (4.7) | 257 (2.9) | < 0.001 |
| Partner's smoking during pregnancy, n (%) | | 8,124 (50.9) | 4,000 (45.2) | < 0.001 |
| Maternal drinking during pregnancy, n (%) | | 329 (2.1) | 181 (2.0) | 0.93 |
| Maternal history of mental disease, n (%) | | 696 (4.4) | 411 (4.6) | 0.31 |
| Maternal history of developmental disorder, n (%) | | 10 (0.06) | 4 (0.04) | 0.58 |
| Maternal history of epilepsy, n (%) | | 69 (0.4) | 54 (0.6) | 0.06 |
| Parity, n (%) | |  |  |  |
|  | Multiparous | 10,298 (64.5) | 5,437 (61.4) | < 0.001 |
| Means of pregnancy for current birth, n (%) | |  |  | < 0.001 |
|  | Spontaneous | 15,033 (94.2) | 8,191 (92.5) |  |
|  | Ovulation induction through medication | 358 (2.2) | 272 (3.1) |  |
|  | Artificial insemination or in vitro fertilization | 573 (3.6) | 396 (4.5) |  |
| Maternal use of folic acid supplements, n (%) | | 298 (1.9) | 168 (1.9) | 0.87 |
| Diabetes mellitus/gestational diabetes mellitus, n (%) | | 523 (3.3) | 299 (3.4) | 0.68 |
| Hypertensive disorder of pregnancy, n (%) | | 414 (2.6) | 265 (3.0) | 0.07 |
| Intrauterine growth restriction, n (%) | | 225 (1.4) | 174 (2.0) | 0.001 |
| Mode of delivery for current birth, n (%) | |  |  | < 0.001 |
|  | Spontaneous delivery | 9,870 (61.8) | 5,116 (57.7) |  |
|  | Induced delivery | 2,777 (17.4) | 1,399 (15.8) |  |
|  | Vacuum extraction/Forceps delivery | 873 (5.5) | 504 (5.7) |  |
|  | Cesarean section | 2,3444 (15.7) | 1,840 (20.8) |  |
| Gestational age (weeks) | | 39 (38, 40) | 39 (38, 40) | < 0.001 ^*^ |
| Gestational age, n (%) | |  |  | < 0.001 |
|  | < 37 weeks | 263 (1.6) | 210 (2.4) |  |
|  | ≥ 37 weeks | 15,701 (98.4) | 8,649 (97.6) |  |
| Birth weight, g | | 3064 (2826, 3312) | 3008 (2764, 3260) | < 0.001 ^*^ |
| Birth weight, n (%) | |  |  | < 0.001 |
|  | < 1,500 g | 4 (0.02) | 3 (0.03) |  |
|  | 1,500-2,499 g | 855 (5.4) | 654 (7.4) |  |
|  | ≥ 2,500 g | 15,105 (94.6) | 8,202 (92.6) |  |
| Gender (male), n (%) | | 7,771 (48.7) | 4,846 (54.7) | < 0.001 |
| Method of feeding, n (%) | |  |  | < 0.001 |
|  | Breast feeding | 9,461 (59.3) | 4,873 (55.0) |  |
|  | Mixed feeding | 5,878 (36.8) | 3,577 (40.4) |  |
|  | Infant formula | 529 (3.3) | 331 (3.7) |  |
|  | Other | 96 (0.6) | 78 (0.9) |  |
| Neonatal jaundice, n (%) | | 1,927 (12.1) | 1,177 (13.3) | 0.006 |
| Total number of positive ASQ-3 domains, n (%) | |  |  | N.A. |
|  | 1 domain | - | 4,905 (55.4) |  |
|  | 2 domains | - | 2,212 (25.0) |  |
|  | 3 domains | - | 1,055 (11.9) |  |
|  | 4 domains | - | 502 (5.6) |  |
|  | 5 domains | - | 185 (2.1) |  |
| BMI, body mass index; GWG, gestational weight gain; JPY, Japanese yen, N.A., not available.  *Mann–Whitney U test of Normal development vs. Positive screen. Continuous variables are expressed as the median (interquartile range).  ^†^The average (median) annual Japanese household income in 2018 was 5,523,000 JPY (4,370,000 JPY). The currency exchange rates on July 12, 2021, were: 1 USD = 110 JPY and 1 EUR = 130 JPY. | | | | |

| **Supplemental table S4. Multivariate logistic regression analysis for risk of delay in ASQ-3 domains versus normal development in children without registered father** | | | | | | | | | | |
| --- | --- | --- | --- | --- | --- | --- | --- | --- | --- | --- |
|  |  | Within GWG (reference) |  | Below GWG | |  | Above GWG | |  | Every 2.3 kg (5 lb) increased |
|  |  | No. cases / Normal development |  | No. cases / Normal development | OR (95% CI) |  | No. cases / Normal development | OR (95% CI) |  | OR (95% CI) |
| ASQ-3 domain | |  |  |  |  |  |  |  |  |  |
|  | Communication | 429/5426 |  | 1149/9159 | 1.42 (1.26-1.61) |  | 98/1379 | 1.03 (0.82-1.31) |  | 0.89 (0.86-0.92) |
|  | Gross motor | 995/5426 |  | 2204/9159 | 1.18 (1.08-1.28) |  | 216/1379 | 0.93 (0.79-1.10) |  | 0.94 (0.91-0.96) |
|  | Fine motor | 679/5426 |  | 1558/9159 | 1.25 (1.13-1.38) |  | 159/1379 | 0.98 (0.81-1.18) |  | 0.93 (0.91-0.96) |
|  | Problem solving | 1095/5426 |  | 2329/9159 | 1.18 (1.08-1.28) |  | 254/1379 | 0.94 (0.81-1.10) |  | 0.95 (0.93-0.98) |
|  | Personal-social | 1259/5426 |  | 2736/9159 | 1.16 (1.07-1.25) |  | 267/1379 | 0.92 (0.79-1.06) |  | 0.94 (0.92-0.97) |

OR, adjusted odds ratio; CI, confidence interval; GWG, gestational weight gain; BMI, body mass index; DM/GDM, diabetes mellitus/gestational diabetes mellitus; HDP, hypertensive disorder of pregnancy.

ORs were adjusted for maternal age, pre-pregnancy BMI, parental smoking habit, maternal drinking habit, maternal highest level of education, annual household income, maternal history of developmental disorders, epilepsy, and mental disease, means of pregnancy, use of folic acid supplements, complications during pregnancy (including DM/GDM and HDP), intrauterine growth restriction, gender, birth weight, method of feeding, and neonatal jaundice.
